# Evaluation of adjuvant psychological therapy for clinically referred cancer patients.

**DOI:** 10.1038/bjc.1991.60

**Published:** 1991-02

**Authors:** S. Greer, S. Moorey, J. Baruch

**Affiliations:** Cancer Research Campaign Psychological Medicine Group, Royal Marsden Hospital, Sutton, Surrey, UK.

## Abstract

Adjuvant psychological therapy (APT) is a newly developed cognitive behavioural treatment which has been designed specifically to improve the quality of life of cancer patients by alleviating emotional distress and inducing a fighting spirit. We report a phase I/II study which evaluates APT in routine clinical practice. A consecutive series of 44 outpatients with various cancers referred for psychiatric consultation and receiving APT at the Royal Marsden Hospital was studied. Standardised self-report questionnaires were used to measure anxiety, depression and four principal categories of mental adjustment to cancer, namely, fighting spirit, helplessness, anxious preoccupation and fatalism. Statistical comparisons between pre-therapy scores and scores after an average of five APT sessions revealed significant improvement in anxiety, depression, fighting spirit, anxious preoccupation and helplessness. Fatalism scores showed the same trend, but the changes were smaller. Patients with advanced disease showed as much improvement as those with local or locoregional disease. Present results indicate improvement in both psychiatric symptoms and mental adjustment to cancer associated with APT. Whether this association is causal remains to be determined by randomised controlled trials. Such a trial is in progress.


Br. J. Cancer (1991), 63, 257-260 © Macmillan Press Ltd., 1991

### Evaluation of adjuvant psychological therapy for clinically referred cancer patients

S. Greer, S. Moorey & J. Baruch

*Cancer Research Campaign Psychological Medicine Group, The Royal Marsden Hospital, Sutton, Surrey SM2 5PT, UK.*

**Summary** Adjuvant psychological therapy (APT) is a newly developed cognitive behavioural treatment which has been designed specifically to improve the quality of life of cancer patients by alleviating emotional distress and inducing a fighting spirit. We report a phase I/II study which evaluates APT in routine clinical practice. A consecutive series of 44 outpatients with various cancers referred for psychiatric consultation and receiving APT at the Royal Marsden Hospital was studied. Standardised self-report questionnaires were used to measure anxiety, depression and four principal categories of mental adjustment to cancer, namely, fighting spirit, helplessness, anxious preoccupation and fatalism. Statistical comparisons between pre-therapy scores and scores after an average of five APT sessions revealed significant improvement in anxiety, depression, fighting spirit, anxious preoccupation and helplessness. Fatalism scores showed the same trend, but the changes were smaller. Patients with advanced disease showed as much improvement as those with local or locoregional disease. Present results indicate improvement in both psychiatric symptoms and mental adjustment to cancer associated with APT. Whether this association is causal remains to be determined by randomised controlled trials. Such a trial is in progress.

Psychological and social morbidity among cancer patients has been well documented in several systematic studies (Morris *et al.*, 1977; Plumb & Holland, 1977; Maguire *et al.*, 1978; Derogatis *et al.*, 1983; Hughes, 1987). Such morbidity may persist for years in long-term survivors even in the absence of any signs of disease (Fobair *et al.*, 1986). Increasing concern about the quality of life of cancer patients has led, recently, to the development of psychological treatment programmes for these patients. Like all treatments in medicine, such psychotherapeutic procedures should be scientifically evaluated by means of randomised controlled trials. The few randomised trials reported so far have produced inconsistent results, reflecting differences in patient populations, in types of psychological treatment and in measures of outcome (Greer, 1989). Certain methodological problems inherent in psychotherapy trials have been identified; though complex and difficult, these problems are not insurmountable (Cawley, 1983; Moorey & Greer, 1989).

The requirements of well-designed trials often lead to study designs which are methodologically rigorous but not directly applicable to clinical practice. Highly specific homogeneous patient samples, screened populations and extensive exclusion criteria may contribute to a marked discrepancy between the experience of patients entering a randomised trial and that obtaining in clinical practice. We are at present conducting a randomised trial of adjuvant psychological therapy (see below). Patients with primary cancers or first recurrence are being screened psychologically by means of standardised questionnaires. Those patients who have high scores indicating psychological morbidity are invited to take part in our trial and, if they agree, are randomised to either therapy or a no therapy control group. Compare this with normal clinical practice at the same hospital: patients who, at medical consultation, appear unduly anxious, depressed or otherwise emotionally distressed are referred by their clinicians to the Psychological Medicine department. Hence, although patients receive adjuvant psychological therapy (APT) whether in the trial or clinically referred, the ways in which they come to therapy differ widely.

In addition to research which evaluates APT in patients discovered to have psychological disturbance through screening methods, there is a place for evaluation of its efficacy in clinically referred patients. Clearly, on ethical grounds it would be undesirable to allocate such patients to a no-treatment control group. As a first step in demonstrating the effectiveness of APT, there is a place for uncontrolled studies of psychological therapy for clinically referred cancer patients. This kind of study should then be followed by randomised controlled trials in which APT is compared with no treatment and, possibly, with other forms of psychological therapy. The present study reports an uncontrolled evaluation of APT in routine clinical practice: anxiety, depression and mental adjustment to cancer among patients referred for psychiatric consultation are compared before and after APT.

### Materials and methods

A consecutive series of out-patients with a confirmed diagnosis of cancer referred for psychiatric consultation at The Royal Marsden Hospital was studied. Patients were entered in the trial: (i) if they were suffering from a formal psychiatric disorder (excluding organic mental disorders, schizophrenia and other psychotic disorders); or (ii) if their psychological disturbance, though not reaching the level of a formal psychiatric disorder, was sufficiently severe to have resulted in more than transient distress. All patients referred during a specified period who fulfilled these criteria were entered in the study.

### *Adjuvant psychological therapy (APT)*

A full description of APT has been provided by Moorey and Greer (1989). APT is a brief structured treatment programme in which the principles of cognitive therapy are applied to the specific problems of cancer patients. Cognitive therapy aims to alleviate emotional disorders by identifying and correcting maladaptive thinking (Beck, 1976). Applied to cancer-related psychological disorders, it is hypothesised that these disorders depend not only on the physical effects of the disease process but also on two crucial factors: (1) the personal meaning of the disease, i.e. how the patient perceives cancer and its implications; and (2) the patient's coping ability, i.e. what the patient thinks and does to reduce the threat posed by cancer. These factors are influenced, in turn, by the degree of emotional support given by family and friends as well as by medical and nursing staff.

APT is focused on these factors. Therapy is directed primarily at current problems and teaches patients new coping skills. APT is conducted with individual patients and, where possible, the spouse. Approximately six sessions, each lasting an hour, are held; occasionally more sessions are

Correspondence: S. Greer.
Received 22 March 1990; and in revised form 8 August 1990.

required. The therapeutic relationship is a collaborative one in which the therapist and patient set an agreed agenda, defining the specific problems to be addressed. These problems are then tackled using various cognitive and behavioural techniques including the following: (a) Patients are taught (i) to identify and record negative automatic thoughts, and (ii) to challenge these thoughts by reality testing; in this way, the negative thoughts can be replaced by more realistic, adaptive coping responses. (b) Patients are encouraged to rehearse, in imagination and role play, impending stressful events and to practise ways of coping with such events. (c) Patients are encouraged to plan and carry out various activities which give both a sense of mastery or control over some aspects of their lives and a sense of pleasure. (d) Relaxation training is used if anxiety is severe. (e) Patients are encouraged to express feelings openly. Frank mutual communication of feelings between the patient and spouse is encouraged in sessions. (f) The personal strengths of the patient are identified and fostered as a means of raising self-esteem, overcoming feelings of helplessness and inducing a fighting spirit. (g) When the patient's predominant reaction to cancer is avoidance (denial) this is not challenged. The disease is not discussed; instead, therapy is focused on any symptoms present and on developing coping skills which will enable the patient to resume normal life as quickly as possible.

### *Measures of outcome*

*Anxiety and Depression* were measured using the Hospital Anxiety and Depression (HAD) scale. This self-rating scale is designed to detect states of anxiety and depression in patients with physical illnesses (Zigmond & Snaith, 1983). It has the advantage that somatic items are excluded as far as possible, so that depression scores are not affected by symptoms such as weight loss and anorexia which are frequently associated with cancer itself.

*Adjustment to cancer* refers to the patient's perception of the implications of cancer and his or her coping strategies, i.e. what the patient thinks and does to reduce the threat posed by cancer. Previous work by our research group has shown that adjustment to cancer can be grouped in the following major categories: fighting spirit, helplessness, anxious preoccupation, fatalism and avoidance (denial). We have developed a self-rating questionnaire, the Mental Adjustment to Cancer (MAC) scale, which measures the first four of these psychological responses (Greer & Watson, 1987; Watson *et al.*, 1988).

The HAD and MAC self-rating scales were completed by patients before therapy and 8 weeks later. Relevant demographic and clinical data including diagnosis, stage of disease, physical performance status (WHO, 1979) and treatments were recorded. A broad staging classification which subsumes all types of cancer seen in this study was used: stage I, local disease only; stage II, locoregional disease, i.e. lymph node involvement (lymphoma grades I and II are included here); stage III, distant metastases (lymphoma grades III and IV and systemic malignant diseases such as leukaemia and myeloma are included here).

### Results

Forty-four consecutive patients who received at least two sessions of APT were assessed before APT and 8 weeks later. The demographic and clinical characteristics of the patients are shown in Table I.

### *Psychological therapy (APT)*

During the 8 weeks' interval between psychological assessments, patients received two to eight (mean 4.55) sessions of APT. At the final 8 weeks assessment, 21 patients (48%) were judged to have completed therapy and discharged; the remaining patients received several more APT sessions in the ensuing weeks. All results here refer to the psychological status of patients 8 weeks after commencing APT, irrespective of whether APT had been completed. In addition to APT, five patients received anti-depressant drugs.

**Table I** Patient characteristics

| | | *Cancer site* | |
|---|---|---|---|
| *Age (years)* | | Breast | 17 (39%) |
| Mean | 47.9 | Non-Hodgkin's lymphoma | 7 (16%) |
| Range | 17–77 | | |
| | | Head and neck | 3 (7%) |
| *Sex* | | G-I tract | 2 (5%) |
| Male | 14 (32%) | Gynaecological | 2 (5%) |
| Female | 30 (68%) | Lung | 2 (5%) |
| | | Melanoma | 2 (5%) |
| *Marital state* | | Myeloma | 2 (5%) |
| Single | 8 (18%) | Prostate/bladder | 2 (5%) |
| Married/cohabiting | 29 (66%) | Other | 4 (9%) |
| Separate/divorced | 6 (14%) | | |
| Widowed | 1 (2%) | *Stage of disease* | |
| | | Local only | 12 (27%) |
| *Performance status* | | Loco-regional | 16 (36%) |
| 0 | 22 (50%) | Metastatic | 16 (36%) |
| 1 | 15 (34%) | | |
| 2 | 7 (16%) | *Primary/recurrent disease* | |
| 3 | 0 | Primary | 25 (57%) |
| 4 | 0 | Recurrent | 19 (43%) |

Total no. of patients = 44.

### *Psychological outcome*

Statistical comparisons between the pre-therapy and final (8 weeks) mean scores on the HAD and MAC scales were carried out, using two-tailed paired $t$ tests. Significant differences were found, i.e. reductions in anxiety, depression, helplessness, anxious preoccupation and fatalism and a significant increase in fighting spirit (Tables II and III).

In order to examine the effect on psychological outcome of APT alone, comparisons between initial and final mean scores were repeated excluding the five patients who had received anti-depressant medication. The same results as described for the whole sample were obtained.

### *Clinical significance of changes in HAD scores*

HAD scores range from 0 to 21 for anxiety and for depression. Scores from 0 to 7 indicate normal levels, 8 to 10 are regarded as borderline and 11 to 21 indicate severe anxiety or depression, i.e. psychiatric disorder (Zigmond & Snaith, 1983). The distributions of patients in each category before therapy and at final assessment are shown in Table IV.

For the purposes of statistical analysis, patients with HAD scores in the normal range (0–7) were compared with patients scoring in the borderline and severe ranges (8–21) before therapy and at final assessment. Table V shows statistically significant reductions in the proportions of patients with high anxiety and depression scores at final assessment. No patient became worse.

**Table II** Changes in HAD scores by psychological therapy (APT)

| | *Pre-APT score mean (s.d.)* | *Final score mean (s.d.)* | *P* |
|---|---|---|---|
| Anxiety | 11.8 (4.2) | 8.0 (3.6) | $<0.001$ |
| Depression | 8.4 (5.3) | 5.4 (4.3) | $<0.001$ |

**Table III** Changes in MAC scores by psychological therapy (APT)

| | *Pre-APT score mean (s.d.)* | *Final score mean (s.d.)* | *P* |
|---|---|---|---|
| Fighting spirit | 46.1 (6.9) | 49.8 (5.7) | $<0.001$ |
| Helplessness | 12.7 (4.2) | 9.6 (2.9) | $<0.001$ |
| Anxious preoccupation | 26.2 (3.5) | 24.2 (4.4) | $=0.0012$ |
| Fatalism | 17.5 (3.7) | 16.6 (4.2) | $=0.04$ |

**Table IV** Degree of anxiety and depression by APT

| | *Anxiety score* | | | |
|---|---|---|---|---|
| | *Normal 0–7* | *Borderline 8–10* | *Severe 11–21* | *Total* |
| Pre-APT (*n*) | 7 (16%) | 8 (18%) | 29 (66%) | 44 (100%) |
| Final assessment (*n*) | 22 (50%) | 13 (30%) | 9 (20%) | 44 |
| | | *Depression* | | |
| Pre-APT (*n*) | 22 (50%) | 9 (20%) | 13 (30%) | 44 |
| Final assessment (*n*) | 33 (75%) | 6 (14%) | 5 (11%) | 44 |

Another way of expressing the results is as follows: (a) Before therapy, 21 patients had high anxiety *and* depression scores, 17 patients had high scores on *either* anxiety or depression and six patients had normal anxiety and depression scores. (b) At final assessment, 11 patients had high anxiety *and* depression scores, 12 patients had high scores on *either* anxiety or depression and 21 patients had normal anxiety and depression scores.

### *Predictors of psychological outcome*

*Multiple regression analyses* Two step-wise multiple regression analyses were carried out with nine possible predictors of outcome: age, sex, primary versus recurrent disease, stage of disease, performance status, number of APT sessions, undergoing chemotherapy or radiotherapy currently (i.e. during the last month), side-effects of such therapy, pre-APT anxiety and depression scores. The two dependent variables were anxiety and depression scores at 8 weeks. The results may be summarised as follows. (i) Anxiety scores at 8 weeks: pre-APT anxiety scores predicted 40% of the variance. (ii) Depression scores at 8 weeks: pre-APT depression scores predicted 50.9% of the variance; the addition of age increased the predicted variance to 59%. (iii) No other variable contributed a significant proportion of the variance.

*Analyses of co-variance* To test further the influence of patient and disease characteristics on psychological outcome, a series of analyses of co-variance was performed using generalised linear modelling. One-way ANCOVAs were performed on assessment at 8 weeks for anxiety and depression scores, with pre-therapy scores entered as co-variates.

(i) Anxiety scores at 8 weeks: no effects were found for sex ($F = 0.32$; d.f. = 1; $P = 0.58$), performance status ($F = 0.58$; d.f. = 1; $P = 0.45$), breast cancer versus other diagnoses ($F = 0.00$; d.f. = 1; $P = 0.96$), presence of symptoms referrable to chemo/radiotherapy, primary versus recurrent disease ($F = 0.40$; d.f. = 1; $P = 0.53$). Stage of disease just failed to reach significance ($F = 2.97$; d.f. = 2; $P = 0.06$).

(ii) Depression scores at 8 weeks: no effects were found for sex ($F = 0.02$; d.f. = 1; $P = 0.88$), performance status ($F = 0.54$; d.f. = 1; $P = 0.47$), breast cancer versus other diagnoses ($F = 0.38$; d.f. = 1; $P = 0.54$), presence of symptoms referrable to chemo/radiotherapy ($F = 0.49$; d.f. = 2; $P = 0.62$), primary versus recurrent disease ($F = 2.69$; d.f. = 1; $P = 0.11$), or disease stage ($F = 0.40$; d.f. = 1; $P = 0.675$).

No significant effects of interaction were found for any of these analyses.

**Table V** Significance of changes in anxiety and depression

| | | *Final Assessment* | | |
|---|---|---|---|---|
| | *HAD scores* | *Normal n* | *Borderline/ severe n* | *Significance*[a] |
| *Anxiety* | | | | |
| Pre-APT | Normal | 7 | 0 | |
| | Borderline/ severe | 16 | 21 | <0.001 |
| *Depression* | | | | |
| Pre-APT | Normal | 22 | 0 | |
| | Borderline/ severe | 11 | 11 | = 0.03 |

[a]McNemar test for significance of change.

### Discussion

The introduction of psychological therapy as part of the overall medical management of patients with cancer is a recent and, in our view, overdue innovation. Its aim is to measurably improve the quality of life of patients with cancer. In order to achieve that aim, alleviation of distressing psychological ill-health is as necessary as, for example, alleviation of pain. We have developed a systematic psychological therapy programme, APT, which needs to be evaluated. The present phase I/II study was undertaken to ascertain the feasibility of conducting APT in a busy cancer hospital, to determine the effect of APT on anxiety, depression and mental adjustment to cancer and, lastly, to identify any clinical predictors of psychological outcome.

Is psychological therapy feasible in a cancer hospital or oncology department? In our experience this will depend on two practical conditions. First, therapy cannot be conducted in hospital wards where conversation can be overheard and interruptions are common. The provision of consulting rooms in a *sine qua non* for psychotherapy. Secondly, the therapists need to adopt a flexible policy regarding appointments, fitting these in wherever possible with appointments to other outpatient and treatment clinics. In this way, patients who are often physically unwell are not burdened with frequent visits to hospital and, equally important, psychological therapy is seen by patients as part of medical treatment.

Flexibility is also required when planning the duration of therapy. Although we aimed to have weekly sessions, several patients could not attend each week either on medical grounds or because they lived a long distance from the hospital; hence, the number of APT sessions during the 8 week assessment period varied from two to eight, the average being five sessions. For obvious reasons, prolonged psychotherapy is neither feasible nor appropriate for most cancer patients. APT has been designed as a short-term therapy. It follows that, if therapy is successful, measurable psychological benefit should occur within a brief period. In the present feasibility study, we have taken 8 weeks after commencement of therapy as the assessment point, irrespective of the number of sessions or whether therapy had been completed.

Our results show a significant reduction in anxiety and depression 8 weeks after APT had been commenced. Outcome was measured by patient self-rating scales in order to obviate bias inherent in clinical assessments by the therapists. The HAD scale was used to measure anxiety and depression. We have found, in a study of 568 patients with cancer, that factor analysis produced two distinct though correlated factors corresponding to the questionnaire's anxiety and depression subscales (Moorey *et al.*, 1991). These results confirm that the separate subscales of the HAD scale should be used. We believe that these studies provide a firm basis for the use of the HAD scale as a measure of anxiety and depression in patients suffering from cancer. In the present series of emotionally distressed patients, 86% were correctly identified by high anxiety or depression scores; the remaining 14% (six patients) scored in the normal range on both subscales.

Statistically significant improvement in anxiety and depression was observed 8 weeks after commencement of APT when patients had received an average of five sessions. At the 8 weeks assessment, nearly half the patients were deemed to have completed therapy. The observed reductions in anxiety and depression were not merely *statistically* significant changes in mean scores but represented *clinically* significant improvement. The proportion of patients with high anxiety scores fell from 84% ($n = 37$) before therapy to 48% ($n = 21$) at final assessment. Depression was less common in our patients, but the same trend was observed: the proportion of patients with high depression scores dropped from 50% ($n = 22$) before therapy to 25% ($n = 11$). It should be noted that no patient became worse as a result of therapy.

Mental adjustment to cancer, measured by the MAC scale, also improved. At 8 weeks assessment, significant decreases in helplessness and anxious preoccupation and a significant increase in fighting spirit were demonstrated. These changes have a beneficial effect on quality of life. Active coping strategies, as subsumed under the heading of fighting spirit, have been shown to be the most effective in managing the stress of breast cancer (Rowland & Holland, 1989). Conversely, helplessness and anxious preoccupation are correlated with depression and anxiety (Watson et al., 1988).

We found no significant predictors of psychological outcome except, as might be expected, pre-therapy anxiety and depression scores; high pre-therapy scores were correlated with high scores at 8 weeks. It is worthy of note that neither disease stage nor performance status predicted psychological outcome suggesting that patients with metastatic as well as local and locoregional disease can benefit from APT. It should be noted, however, that the present patient sample did not include any severely disabled patients, i.e. those with WHO performance status 3 and 4.

The present study has established the feasibility of APT in a cancer hospital and demonstrated significant improvement in anxiety, depression and mental adjustment to cancer following a brief course (averaging five sessions) of APT. This phase I/II study cannot, of course, determine whether APT was responsible for the observed improvement in quality of life. But our results are sufficiently encouraging to mount a controlled trial of APT.

We are grateful to the Cancer Research Campaign for its generous funding, to Ian Fryatt for valuable statistical advice, to Gill Chesney our secretary and data manager, and especially to our patients who worked so hard to get better.

### References

BECK, A.T. (1976). *Cognitive Therapy and the Emotional Disorders.* International Universities Press: New York.

CAWLEY, R.H. (1983). The principles of treatment and therapeutic evaluation. In *Handbook of Psychiatry 1: General Psychopathology*, Shepherd, M. & Zangwill, O. (eds), p. 221. Cambridge University Press: Cambridge.

DEROGATIS, L.R., MORROW, G.R., FETTING, J. & 5 others (1983). The prevalence of psychiatric disorders among cancer patients. *JAMA*, **249**, 751.

FOBAIR, P., HOPPE, R.T., BLOOM, J., COX, R., VARGHESE, A. & SPIEGEL, D. (1986). Psychosocial problems among survivors of Hodgkin's disease. *J. Clin. Oncol.*, **4**, 805.

GREER, S. & WATSON, M. (1987). Mental adjustment to cancer: its measurement and prognostic importance. *Cancer Surv.*, **6**, 439.

GREER, S. (1989). Can psychological therapy improve the quality of life of patients with cancer? *Br. J. Cancer*, **59**, 149.

HUGHES, J.E. (1987). Psychological and social consequences of cancer. *Cancer Surv.*, **6**, 455.

MAGUIRE, G.P., LEE, E.G., BEVINGTON, D.J., KUCHEMAN, C.S., CRABTREE, R.J. & CORNELL, C.F. (1978). Psychiatric problems in the first year after mastectomy. *Br. Med. J.*, **i**, 963.

MOOREY, S. & GREER, S. (1989). *Psychological Therapy for Patients with Cancer: a New Approach.* Heinemann Medical Books: Oxford.

MOOREY, S., GREER, S., WATSON, M. & 5 others (1991). The factor structure and factor stability of the Hospital Anxiety and Depression scale in patients with cancer. *Br. J. Psychiatr.* (in the press).

MORRIS, T., GREER, H.S. & WHITE, P. (1977). Psychological and social adjustment to mastectomy: a two-year follow-up study. *Cancer*, **40**, 2381.

PLUMB, M. & HOLLAND, J.C. (1977). Comparative studies on psychological functions in patients with advanced cancer: I. Self-reported depressive symptoms. *Psychosom. Med.*, **39**, 264.

ROWLAND, J.H. & HOLLAND, J.C. (1989). Breast cancer. In *Handbook of Psychooncology*, Holland, J. & Rowland, J. (eds), p. 193. Oxford University Press: Oxford.

WATSON, M., GREER, S., YOUNG, J., INAYAT, Q., BURGESS, C. & ROBERTSON, B. (1988). Development of a questionnaire measure of adjustment to cancer: the MAC scale. *Psychol. Med.*, **18**, 203.

WORLD HEALTH ORGANIZATION (1979). *WHO Handbook for Reporting Results of Cancer Treatment.* WHO: Geneva.

ZIGMOND, A.S. & SNAITH, R.P. (1983). The Hospital Anxiety and Depression scale. *Acta Psychiat. Scand.*, **67**, 361.


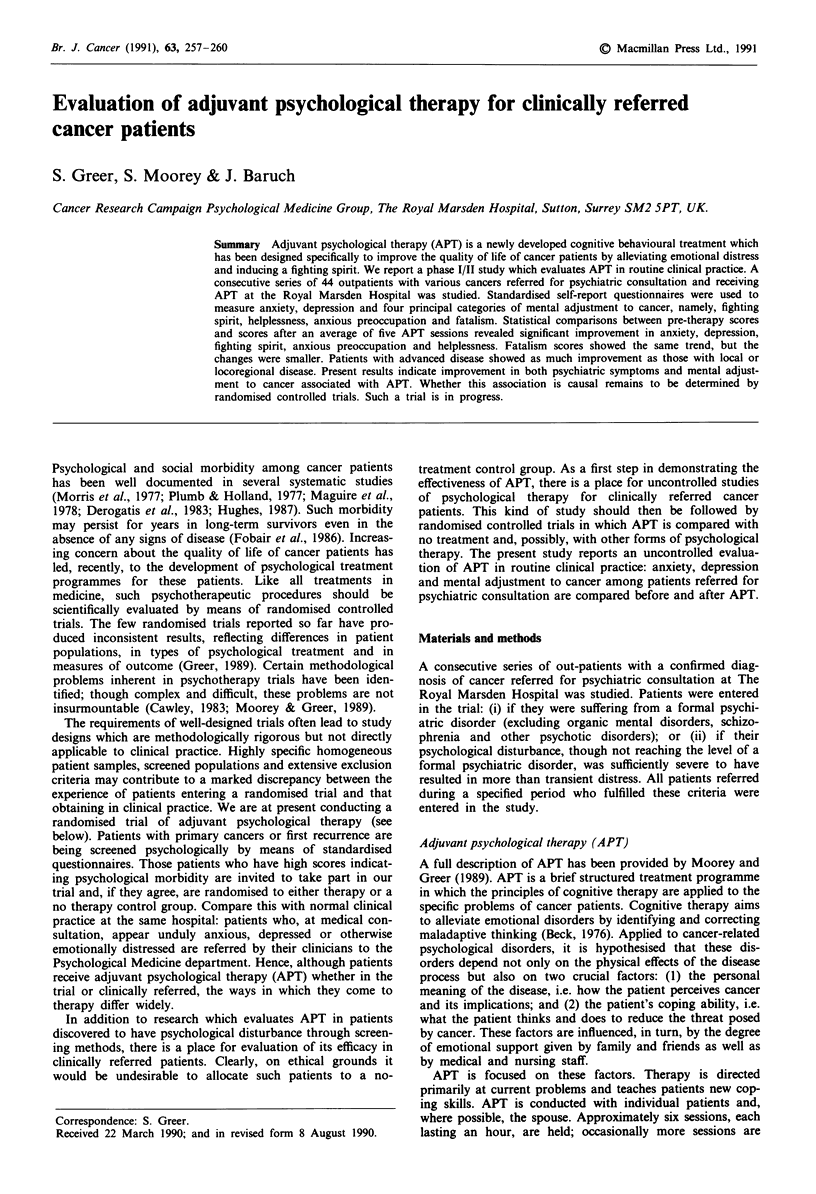

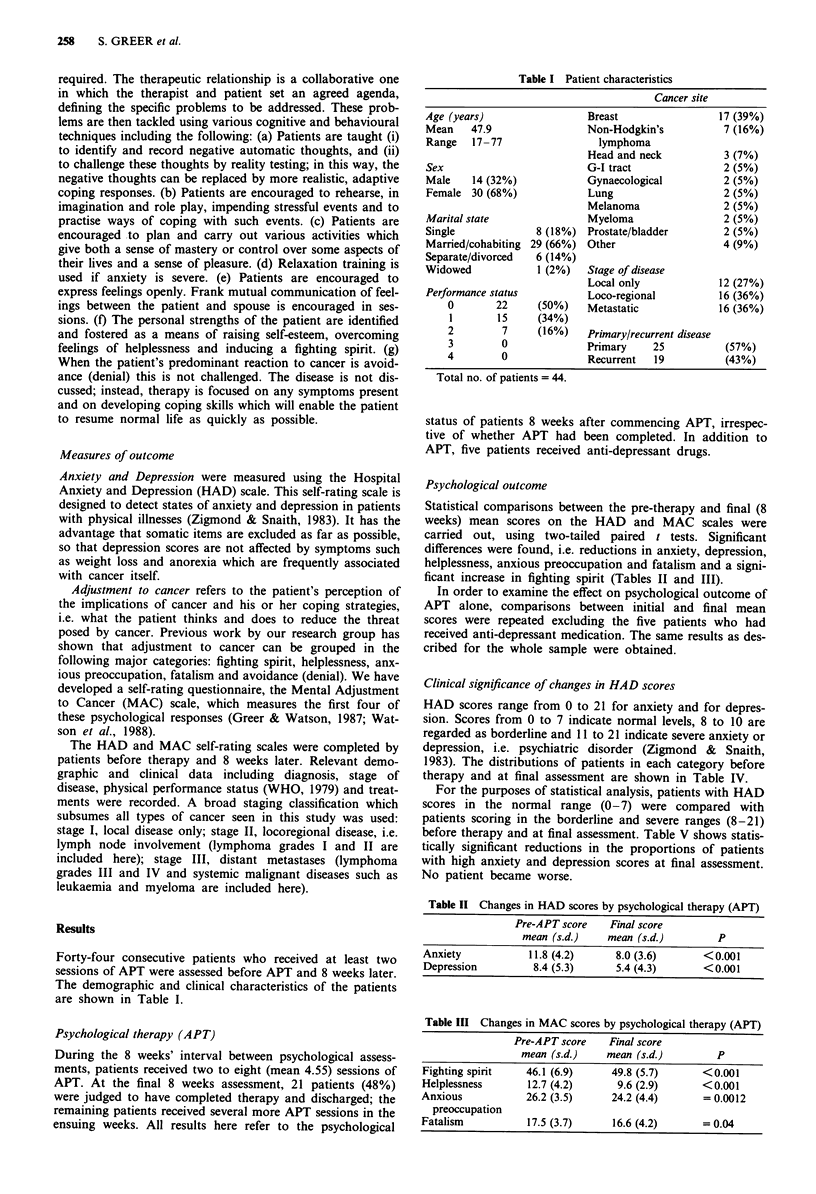

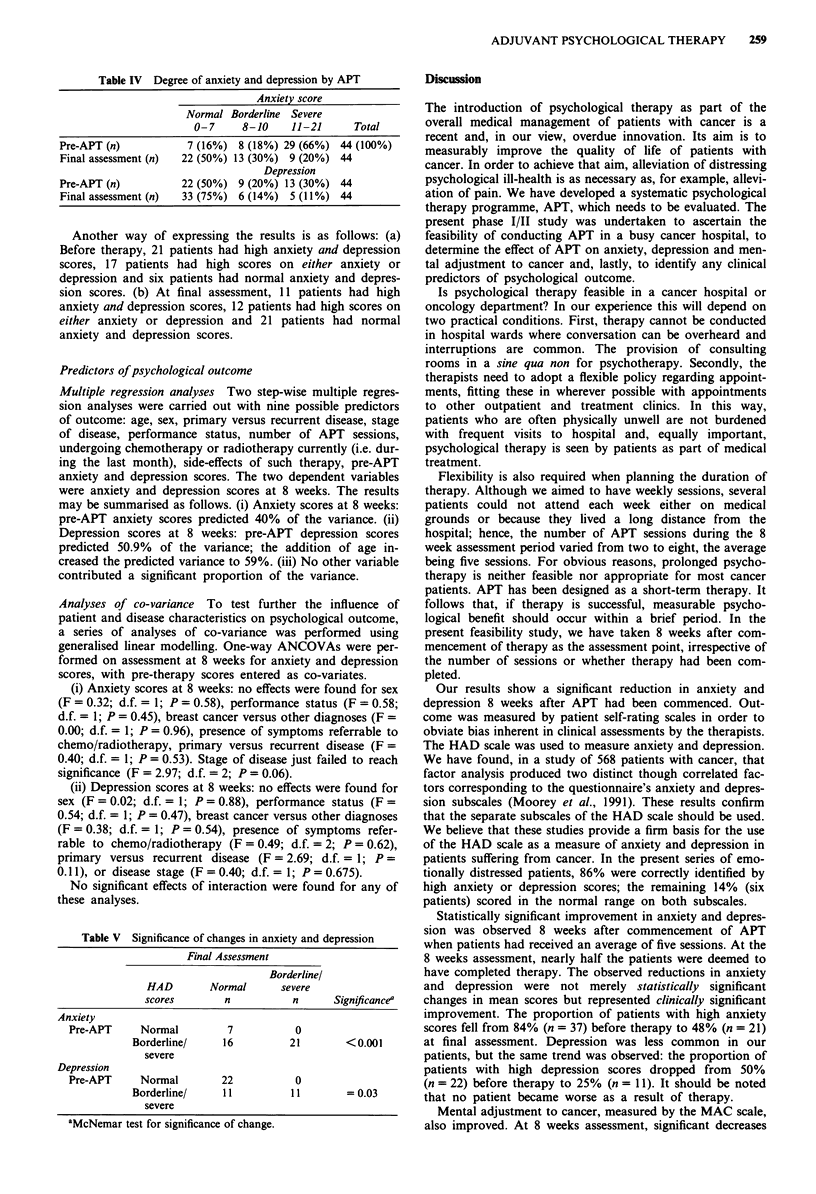

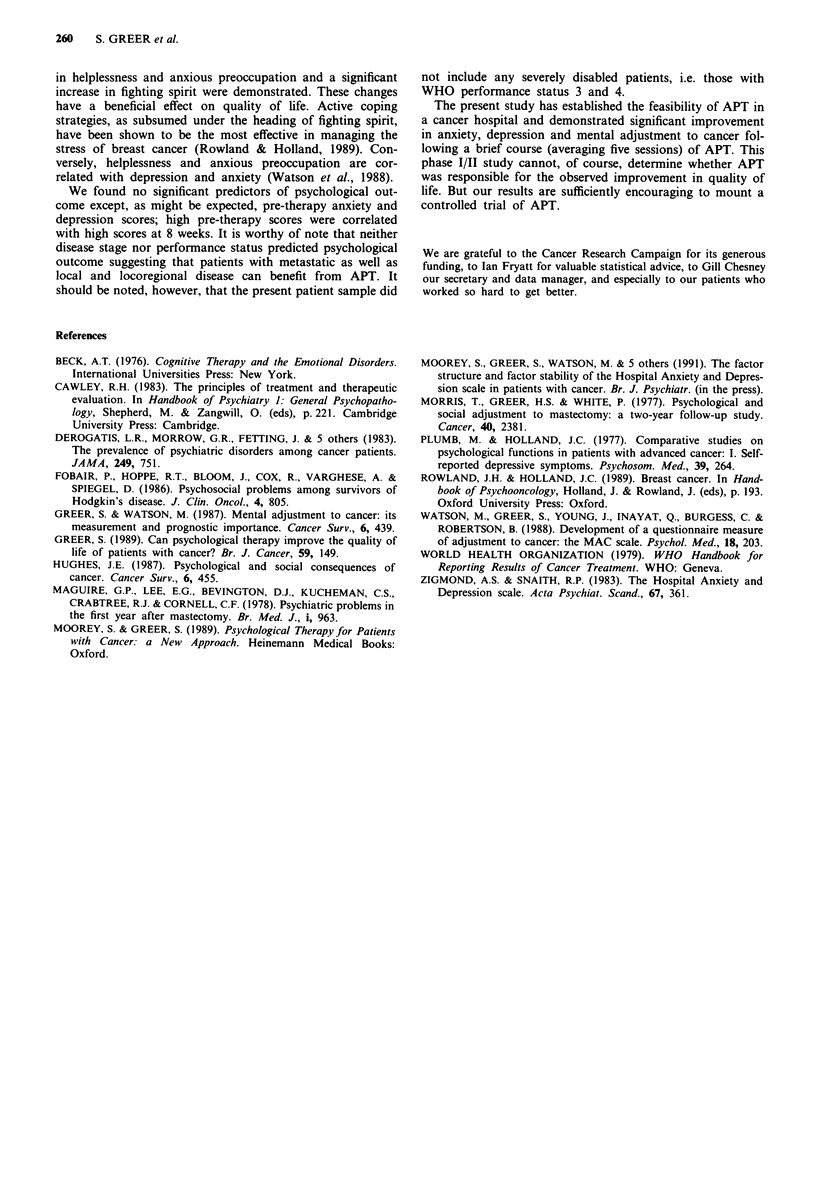


## References

[OCR_00583] Derogatis L. R., Morrow G. R., Fetting J., Penman D., Piasetsky S., Schmale A. M., Henrichs M., Carnicke C. L. (1983). The prevalence of psychiatric disorders among cancer patients.. JAMA.

[OCR_00588] Fobair P., Hoppe R. T., Bloom J., Cox R., Varghese A., Spiegel D. (1986). Psychosocial problems among survivors of Hodgkin's disease.. J Clin Oncol.

[OCR_00596] Greer S. (1989). Can psychological therapy improve the quality of life of patients with cancer?. Br J Cancer.

[OCR_00593] Greer S., Watson M. (1987). Mental adjustment to cancer: its measurement and prognostic importance.. Cancer Surv.

[OCR_00600] Hughes J. E. (1987). Psychological and social consequences of cancer.. Cancer Surv.

[OCR_00604] Maguire G. P., Lee E. G., Bevington D. J., Küchemann C. S., Crabtree R. J., Cornell C. E. (1978). Psychiatric problems in the first year after mastectomy.. Br Med J.

[OCR_00618] Morris T., Greer H. S., White P. (1977). Psychological and social adjustment to mastectomy: a two-year follow-up study.. Cancer.

[OCR_00623] Plumb M. M., Holland J. (1977). Comparative studies of psychological function in patients with advanced cancer--I. Self-reported depressive symptoms.. Psychosom Med.

[OCR_00633] Watson M., Greer S., Young J., Inayat Q., Burgess C., Robertson B. (1988). Development of a questionnaire measure of adjustment to cancer: the MAC scale.. Psychol Med.

[OCR_00641] Zigmond A. S., Snaith R. P. (1983). The hospital anxiety and depression scale.. Acta Psychiatr Scand.

